# Use of health services by Brazilian older adults with and without functional limitation

**DOI:** 10.1590/S1518-8787.2017051000243

**Published:** 2017-06-01

**Authors:** Alexandre Moreira de Melo Silva, Juliana Vaz de Melo Mambrini, Sérgio Viana Peixoto, Deborah Carvalho Malta, Maria Fernanda Lima-Costa

**Affiliations:** I Programa de Pós-Graduação em Saúde Coletiva. Centro de Pesquisas René Rachou. Fundação Oswaldo Cruz. Belo Horizonte, MG, Brasil; II Núcleo de Estudos em Saúde Pública e Envelhecimento. Centro de Pesquisas René Rachou. Fundação Oswaldo Cruz. Belo Horizonte, MG, Brasil; III Departamento de Enfermagem Aplicada. Escola de Enfermagem. Universidade Federal de Minas Gerais. Belo Horizonte, MG, Brasil; IV Departamento de Enfermagem Materno Infantil e Saúde Pública. Escola de Enfermagem. Universidade Federal de Minas Gerais. Belo Horizonte, MG, Brasil

**Keywords:** Aged, Frail Elderly, Disabled Persons, Activities of Daily Living, Health Services for the Aged, Quality of Health Care, Idoso, Idoso Fragilizado, Pessoas com Deficiência, Atividades Diárias, Serviços de Saúde para Idosos, Qualidade da Assistência à Saúde

## Abstract

**OBJECTIVE:**

To analyze the use of health services and the quality of medical care received by Brazilian older adults with and without functional limitation.

**METHODS:**

The main analyses were based on a national sample representing 23,815 participants of the National Survey on Health (PNS) aged 60 years or older. Functional limitation was defined by the difficulty to perform at least one out of ten basic or instrumental activities of daily living. Potential confounding variables included predisposing and enabling factors of the use of health services.

**RESULTS:**

The prevalence of functional limitation was 30.1% (95%CI 29.2–31.4). The number of doctor visits and hospitalizations in the past 12 months showed statistically significant associations with functional limitation, both for users of the public system (OR = 2.48 [95%CI 2.13–2.88] for three or more doctor visits and OR = 2.58 [95%CI 2.15–3.09] for one or more hospitalizations) and of the private system (OR = 2.56 [95%CI 1.50–4.36] and OR = 2.22 [95%CI 1.64–3.00], respectively). The propensity to use basic health units was higher among users of the private system with functional limitations (OR = 2.01 [95%CI 1.12–3.59]). Only two out of seven indicators of the quality of medical care received were associated with functional limitation, in the perception of users of public and private systems. The public system users with functional limitations did worse evaluation of the freedom for choosing the doctor and waiting time for an appointment, when compared with users of the same system without these limitations (OR = 0.81 [95%CI 0.67–0.99] and OR = 0.76 [95%CI 0.62–0.93], respectively).

**CONCLUSIONS:**

Older adults with functional limitations use more health services in comparison with those without such limitations. The magnitude of the association between functional limitation and number of doctor visits and hospitalizations was similar in the public and private health systems.

## RESUMO

**OBJETIVO:**

Analisar o uso de serviços de saúde e a qualidade da atenção médica recebida por idosos brasileiros com e sem limitação funcional.

**MÉTODOS:**

As análises principais foram baseadas em amostra nacional representativa de 23.815 participantes da Pesquisa Nacional de Saúde com 60 anos ou mais. A limitação funcional foi definida pela dificuldade para realizar pelo menos uma entre dez atividades básicas ou instrumentais da vida diária. Potenciais variáveis de confusão incluíram fatores predisponentes e facilitadores do uso de serviços de saúde.

**RESULTADOS:**

A prevalência da limitação funcional foi de 30,1% (IC95% 29,2−31,4). O número de consultas médicas e a ocorrência de hospitalizações nos últimos 12 meses apresentaram associações estatisticamente significantes com a limitação funcional, tanto entre usuários do sistema público (OR = 2,48 [IC95% 2,13−2,88] para três ou mais consultas e OR = 2,58 [IC95% 2,15−3,09] para uma ou mais hospitalizações) quanto do sistema privado (OR = 2,56 [IC95% 1,50−4,36] e OR = 2,22 [IC95% 1,64−3,00], respectivamente). A propensão ao uso da unidade básica de saúde foi maior entre usuários do sistema privado com limitações funcionais (OR = 2,01 [IC95% 1,12−3,59]). Entre sete indicadores da qualidade da atenção médica recebida, na percepção dos usuários dos sistemas público e privado, somente dois apresentaram associação com a limitação funcional. Os usuários do sistema público com limitações funcionais fizeram pior avaliação da liberdade para escolha do médico e do tempo de espera para a consulta, em comparação aos usuários do mesmo sistema sem essas limitações (OR = 0,81 [IC95% 0,67−0,99] e OR = 0,76 [IC95% 0,62−0,93], respectivamente).

**CONCLUSÕES:**

Idosos com limitações funcionais utilizam mais serviços de saúde em comparação aos seus pares sem essas limitações. A magnitude da associação entre a limitação funcional e o número de consultas médicas e de hospitalizações foi semelhante nos sistemas público e privado de saúde.

## INTRODUCTION

The aging of the population occurs worldwide, and Brazil – the fifth largest population in the world – is one of the countries where this demographic transition has been occurring more rapidly^a^. Aged populations have a higher burden of chronic diseases and disabilities, leading to increased demand for health care. There is, therefore, growing interest in analyzing the profile and inequalities associated with the use of health services by older adults with functional limitations, both in countries with aged populations and in those with rapid aging process^1-3^.

The determinants of use of health-care services are related to contextual factors (types of health systems and their organization, for example)^4^ and individual factors. According to the classical model of Andersen and Newman^5^, individual factors comprehend predisposing characteristics (such as age and sex), enabling characteristics (such as education level and income), and health needs. In Brazil, the organization of health care is based on the Brazilian Unified Health System (SUS), which is responsible for the universal and free provision of health services and programs^6^. The public system coexists with the private one, which requires payment to access it^6^. Adults covered by private health insurances perform more medical and dental appointments in comparison to the rest of the population^7^. In the perception of the adult population, the main differences between the care received in the public health system and that received in the private one are: how to schedule a doctor visit (with predominance of prescheduling in the latter); the waiting time to get the doctor visit (longest in the former); the type of doctor (predominance of general practitioner in the former); and the reason for the doctor visit (predominance of periodic health examination in the latter)^8^.

The prevalence of functional limitation varies between countries and on the basis of the criterion adopted for its definition^9,b^. A widely used definition is the report of difficulties to perform basic or instrumental activities of daily living. Based on this definition, the prevalence of functional limitations in the population aged 50 years or older ranges between 25% in Spain and England and 40% in the United States^10^.

In Brazil, population-based studies examining the association between functional limitation and use of health services are scarce. A study conducted in cities of South and Northeast regions showed that, among older adults with chronic diseases, the prevalence of doctor visits was 30% higher among those with some level of functional limitation, when compared with those without such limitations^11^. Another study, conducted in the metropolitan region of Belo Horizonte, showed that functional limitations were associated with increased hospitalizations and doctor visits at the household^12^.

In this study, we used data from the National Survey on Health (PNS) to analyze the use of health services and the quality of medical care received in the perception of Brazilian older adults with and without functional limitation.

## METHODS

The PNS was held in 2013 by the Brazilian Institute of Geography and Statistics (IBGE) in partnership with the Brazilian Ministry of Health^c^. The survey was designed to represent the adult population, based on complex sampling^13,c^. PNS consists of three questionnaires: household; individual to be responded for all residents; individual to be responded by a selected adult resident. All residents in the sampled households aged 60 years or older were eligible to respond to the module on functional capacity. The selected adult resident was eligible to respond to the module on medical care. For this study, all participants from the survey aged 60 years or older were selected for the analysis of the factors associated with the use of health services (n = 23,815), and the selected residents in the same age group were selected for the analysis of the indicators of quality of medical care received (n = 9,290).

The structure of the PNS questionnaire consists of separate questions on the degree of difficulty to perform different activities of daily life, with response options ranging between no difficulty, little difficulty, great difficulty, and cannot perform it at all. This analysis considered six basic activities (feeding; bathing; using the toilet; dressing; walking at home from one room to another on the same floor; and lie down or get up from bed) and four instrumental activities of daily living (shopping; managing own finances; taking medications; and leaving home using transportation). Functional limitation was assigned to those who reported some degree of difficulty to perform at least one of the above mentioned activities.

The source of care was categorized into public system users and private system users. The latter were defined by the report of having private health plan, from company or public agency, except for exclusively dental plan. The use of the public system was assigned to those who did not have private health plan, even if, possibly, it has paid all or part of the received care.

Five indicators of use of health services were considered: number of doctor visits performed in the past 12 months; occurrence of one or more hospitalizations in the past 12 months; specialty of the doctor in the last appointment (generalist versus specialist); existence of a service or doctor of reference; and location usually sought for health care, among those who reported having service or doctor of reference. The number of doctor visits was categorized as above (three or more doctor visits) and below the median. The condition of having a reference doctor or service was assigned to those who reported having a service or doctor that they usually seek when needing health care. The location of the search was categorized as basic health unit (UBS); doctor’s office or private clinic; emergency room services (emergency care or first aid units of public or private hospital); and elsewhere.

The quality of the medical care received was defined by the user rating regarding the last doctor visit. Seven indicators were considered: physician’s ability to treat the study participants; physician’s respect in the way of serving them; clarity in the physician’s explanations; availability of time for asking questions about the problem or treatment; possibility to talk in privacy with the doctor; freedom to choose the doctor; and waiting time for the appointment. In the PNS questionnaire, the questions about the perception of medical care had five response options, ranging from very good to very bad. In this analysis, responses were categorized into very good or good versus regular, bad, or very bad.

The selection of potential confounding variables for this analysis was based on the theoretical model of Andersen and Newman^5^, considering predisposing and enabling factors for the use of health services. Among the predisposing factors, sex and age (continuous variable) were considered. Among the enabling factors, living with spouse/partner (yes or no) and education level (categorized into five groups, ranging from no education to high school or more) were considered.

The response variables were the indicators of use of health services and of the quality of medical care received, as described above. The exposure of interest was the functional limitation, as previously defined.

Bivariate analysis was based on prevalence estimates and 95% confidence intervals. Linear regression and Pearson’s Chi-square test were used to examine the statistical significance of the differences between means and proportions, respectively.

The multivariate analysis of the association between functional limitation and indicators of use of health services and quality of medical care was based on odds ratio (OR) estimates by binary or multinomial logistic regression^14,15^. The latter was used when the response variable had more than two categories, as was the case of the location sought for health care (four categories) and number of doctor visit (three categories). Binary logistic regression was used in the other situations. The multivariate models were adjusted for age, sex, living with spouse/partner, and education level. The analyses were stratified by the source of health care (public and private).

Multinomial logistic regression and binary logistic regression were used to estimate, respectively, the predicted probability of occurrence of three or more doctor visits and one or more hospitalizations in the past 12 months, according to functional limitation and source of health care.

All analyses were performed using the procedures for complex samples of the statistical package Stata (version 13.0), considering the individual weights and sampling parameters.

PNS was conducted in conformity with the parameters set out in the Declaration of Helsinki. The survey was approved by the National Human Subject Research Ethics Committee (CONEP – Process: 328,159, June 26, 2013)^13^.

## RESULTS

Among the 23,815 older adults who took part in the sample, 29.2% (95%CI 27.8–30.7) had private health insurance and 30.1% presented functional limitation. The prevalence of functional limitation was significantly higher among users of the public system (32.2%) when compared with those of the private system (25.1%). More details on the sociodemographic characteristics of the survey participants and their distributions according to source of health care can be seen in Table 1.

**Table 1:** Sociodemographic characteristics of sample participants aged 60 years or older, according to source of health care. National Survey on Health, 2013. (n = 23,815).

Characteristic	Total		Source of health care				p^b^
	
	Mean or percentage^a^	(95%CI)	Private		Public		
	
			Mean or percentage^a^	(95%CI)	Mean or percentage^a^	(95%CI)	
Age, mean	69.9	69.7–70.1	70.2	69.8–70.6	69.8	69.5–70.0	< 0.001
Female sex, %	56.4	42.8–44.4	59.9	58.5–61.3	54.9	54.0–55.9	< 0.001
Living with spouse/partner, %	57.0	55.9–58.2	59.8	57.5–62.0	55.9	54.6–57.2	< 0.003
Education level, %							
Has never studied	31.8	30.5–33.0	12.8	11.3–14.6	39.6	38.1–41.0	< 0.001
Some elementary or middle school	39.3	38.1–40.5	35.2	32.8–37.7	41.0	39.6–42.4	
Elementary or middle school/some high school	8.3	7.7–9.0	10.1	8.9–11.4	7.6	6.9–8.3	
High school or more	20.6	19.5–21.9	41.9	39.3–44.6	11.9	11.0–12.8	< 0.001
Functional limitation^c^, %	30.1	29.2–31.4	25.1	23.4–26.9	32.2	31.1–33.4	< 0.001

^a^ Means and percentages estimated considering the individual weights and sampling parameters.

^b^ Pearson’s Chi-square test for differences between frequency and linear regression for difference between the means.

^c^ Difficulty to perform one or more basic or instrumental activities of daily living.

Table 2 presents the results of the bivariate analysis of the association between functional limitation, indicators of use of health services, and indicators of quality of medical care received, according to source of health care. The number of doctor visits and the occurrence of one or more hospitalizations in the past 12 months showed statistically significant associations (p < 0.05) with functional limitations, among users of both public and private health systems. Users of the public system reported higher proportion of care by general practitioner, regardless of the functional limitation, in comparison to users of the private system. In the latter, the percentage of care by this professional was significantly higher among older adults with functional limitations when compared with those without such limitations. The UBS was the main place sought for health care among public system users with and without functional limitation, while, in both groups, the doctor’s office or private clinic was the predominant location of search by older adults covered by private health insurance. When comparing those with and without functional limitations, the increased demand for UBS was significantly higher among private system users with limitations (18.2%) when compared with users of the same system without limitations (9.8%). In bivariate analysis, other indicators of use of services and quality of care received did not show statistically significant associations with functional limitation. However, we highlight that, in most indicators of satisfaction with the quality of medical care received, the prevalence of those who assessed these criteria as good or very good was above 75% for most indicators analyzed in both public and private systems. The lowest prevalence was for freedom for choosing the doctor and waiting time, which were lower among users of the public system.

**Table 2:** Bivariate analysis of the association between functional limitationa, indicators of use of health services, and indicators of medical care quality in the last doctor visit carried out among sample participants aged 60 years or older, according to source of health care. National Survey on Health, 2013.

Indicator	Public				Private			
	
	With limitation		Without limitation		With limitation		Without limitation	
			
	%^b^	95%CI^b^	%^b^	95%CI^b^	%^b^	95%CI^b^	%^b^	95%CI^b^
Use of health services (n = 23,815)								
Number of doctor visits in the past 12 months								
None	12.6	11.4–13.9	23.2	22.0–24.4^c^	4.8	3.0–7.4	9.9	8.4–11.6^c^
1–2	24.0	22.3–25.8	31.5	30.1–32.9	20.9	17.6–24.8	33.9	31.3–36.6
≥ 3	63.4	61.4–65.4	45.4	43.8–47.0	74.3	70.2–78.1	56.2	53.4–59.0
One or more hospitalizations in the past 12 months	16.2	14.6–18.0	6.4	5.7–7.1^c^	20.3	16.9–24.1	9.2	7.7–11.0^c^
Medical care by general practitioner in the last doctor visit^d^	65.7	63.9–70.9	68.3	65.7–70.7	38.5	31.8–45.6	28.9	25.3–32.8^c^
Has health service or doctor of reference	79.1	77.2–80.9	76.9	75.3–78.4	85.3	81.5–88.0	82.9	80.5–85.1
Location usually sought (among those who have health service or doctor of reference)								
Basic health unit	59.0	56.1–61.9	62.4	60.3–64.5^c^	18.2	14.2–23.1	9.8	8.0–11.9^c^
Doctor’s office or private clinic	7.8	6.6–9.2	8.9	7.7–10.2	54.3	49.0–59.5	59.4	55.6–63.2
Emergency room service	13.5	11.7–15.6	10.7	9.5–12.2	19.9	15.9–24.6	20.9	17.8–24.4
Other	19.7	17.6–21.9	18.0	16.4–19.6	7.6	5.7–10.1	9.9	7.9–12.3
Quality of medical care on the last doctor visit (very good/good versus regular/bad/very bad) (n = 9,290)								
Physician’s ability to treat them	88.6	86.4–90.5	88.5	86.6–90.2	94.5	92.3–96.1	95.2	92.9–96.8
Physician’s respect in the way of serving them	90.1	87.8–92.0	90.8	89.0–92.4	95.6	92.8–97.3	96.1	94.1–97.4
Clarity in the physician’s explanations	83.2	80.1–86.0	85.9	83.7–87.8	92.5	89.4–94.8	93.3	90.9–95.1
Availability of time for asking questions about the problem	78.7	75.5–81.5	79.7	77.2–82.0	89.2	85.5–92.0	91.3	88.7–93.4
Possibility to talk in privacy with the doctor	83.8	81.0–86.3	84.6	82.4–86.6	91.9	88.8–94.2	92.9	90.3–94.8
Freedom to choose the doctor	56.0	52.3–59.6	58.2	55.2–61.1	77.7	71.1–83.2	83.6	79.8–86.7
Waiting time for the appointment	59.0	55.4–62.5	62.0	59.3–64.7	73.9	67.8–79.2	72.2	67.7–76.3

^a^ Difficulty to perform one or more basic or instrumental activities of daily living.

^b^ Percentages and 95% confidence intervals estimated considering the individual weights and sampling parameters.

^c^ p < 0.05 for differences between the groups with and without functional limitation (Pearson’s Chi-square test).

^d^ General practitioner, family and community doctor, or internist.

Table 3 presents the results of multivariate analysis of the association between functional limitation and the above-mentioned indicators. After adjustments for predisposing and enabling factors, the number of doctor visits in the past 12 months showed strong association with functional limitation both among users of the public system (OR = 1.39; 95%CI 1.18–1.63 for one or two doctor visits and OR = 2.48; 95%CI 2.13–2.88 for three or more doctor visits) and of the private system (OR = 1.20; 95%CI 0.69–2.09 and OR = 2.56; 95%CI 1.50–4.36, respectively). Functional limitation also showed strong association with the occurrence of one or more hospitalizations in both groups (OR = 2.58; 95%CI 2.15–3.09 and OR = 2.22; 95%CI 1.64–3.00, respectively). The previously mentioned association between functional limitation and care by general practitioner in the private system has lost statistical significance in multivariate analysis. The greater use of UBS among private system users with limitations remained after adjustment for these covariates (OR = 2.01; 95%CI 1.12–3.59). Between users of the public system (but not of the private one), the freedom for choosing the doctor and the waiting time presented independent and negative associations with functional limitation (OR = 0.81; 95%CI 0.67–0.99 and OR = 0.76; 95%CI 0.62–0.93, respectively).

**Table 3:** Association between functional limitationa, indicators of use of health services, and indicators of medical care quality in the last doctor visit carried out among sample participants aged 60 years or older, according to source of health care (National Survey on Health, 2013).

Indicators	Public^b^		Private^b^	
	
	OR	95%CI	OR	95%CI
Use of health services (n = 23,815)				
Number of doctor visits in the past 12 months (versus none)				
1–2	1.39	1.18–1.63^c^	1.20	0.69–2.09
≥ 3	2.48	2.13–2.88^c^	2.56	1.50–4.36^c^
One or more hospitalizations in the past 12 months	2.58	2.15–3.09^c^	2.22	1.64–3.00^c^
Medical care by general practitioner in the last doctor visit^d^	0.99	0.82–1.29	0.93	0.62–1.38
Has health service or doctor of reference	1.15	1.00–1.32	1.11	0.82–1.52
Location usually sought (among those with health service or doctor of reference) (versus others)				
Basic health unit	0.88	0.74–1.05	2.01	1.12–3.59^c^
Doctor’s office or private clinic	0.86	0.64–1.14	1.28	0.83–1.97
Emergency room service	1.18	0.93–1.51	1.36	0.79–2.32
Quality of medical care in the last doctor visit (very good/good versus regular/bad/very bad) (n = 9,290)				
Physician’s ability to treat them	0.95	0.73–1.23	0.74	0.38–1.42
Physician’s respect in the way of serving them	0.87	0.65–1.15	0.84	0.41–1.74
Clarity in the physician’s explanations	0.76	0.57–1.00	0.80	0.47–1.37
Availability of time for asking questions about the problem	0.86	0.67–1.11	0.78	0.48–1.28
Possibility to talk in privacy with the doctor	0.87	0.66–1.14	0.73	0.41–1.31
Freedom to choose the doctor	0.81	0.67–0.99^c^	0.64	0.38–1.09
Waiting time for the appointment	0.76	0.62–0.93^c^	0.95	0.63–1.43

^a^ Difficulty to perform one or more basic or instrumental activities of daily living.

^b^ Odds ratio and 95% confidence interval adjusted for age, sex, living with spouse or partner, and education level and estimated by multinomial logistic regression (number of doctor visits and location usually sought) and binary logistic regression (other events); the exposure category was the functional limitation and the response variables were the indicators of use of health services and the quality of medical care received.

^c^ p < 0.05 (Wald test).

^d^ General practitioner, family and community doctor, or internist.

The Figure shows the predicted probability of occurrence of three or more doctor visits and one or more hospitalizations in the different ages, according to functional limitation and source of health care. The probability of occurrence of three or more doctor visits showed clear stratification in all ages, with greater probability among older adults with functional limitations using the private system, followed by those with functional limitations using the public system and by those without functional limitations using private and public systems, respectively. The same stratification was observed for the probability of occurrence of one or more hospitalizations.

**Figure:**
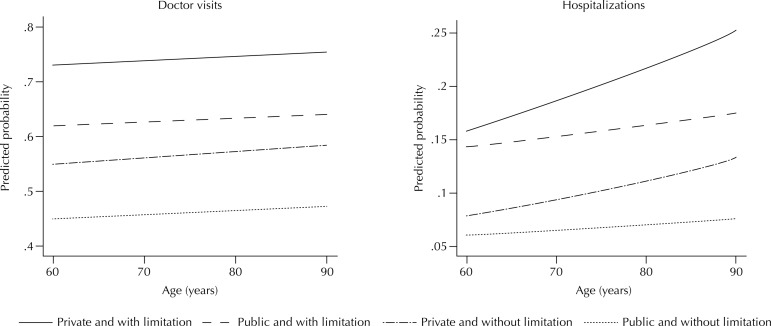
Predicted probabilitya for three or more doctor visits and one or more hospitalizations in the past 12 months along age continuum, according to functional limitationb and source of health care. National Survey on Health, 2013.

^a^ Models adjusted for age, sex, living with spouse or partner, and education level and estimated by multinomial logistic regression (number of doctor visits) and binary logistic regression (hospitalizations).

^b^ Difficulty to perform one or more basic or instrumental activities of daily living.

## DISCUSSION

The results of this study showed that older adults with functional limitations perform more doctor visits and are more prone to occurrence of hospitalizations, regardless of predisposing factors, such as age and sex, and enabling factors, such as living with spouse/partner and education level. We observed strong associations between functional limitation and increased number of doctor visits, as well as between functional limitation and occurrence of one or more hospitalizations, both in the public and private systems.

These results are similar to those from national health surveys that observed association between functional limitation and increased number of doctor visits or hospitalizations in different countries such as United States^1^, China^2^, South Korea^16^, and Taiwan^3^. The main explanation for the increased use of health services by older adults with functional limitations lies in the higher prevalence of chronic diseases and comorbidities (and the risk of complications related to them), which, in turn, lead to the increased use of medications^1,2,17^.

The doctor visit is a positive aspect of health care, for being an opportunity for early diagnosis, prevention, treatment^18^, and referrals to rehabilitation, when appropriate. However, the excessive use of health services is an indicator of care with low solution. Hospitalizations, particularly, can be prevented, if unnecessary. In fact, complications of several diseases can be prevented with effective actions from the primary health care^19^. A Brazilian study, based on about 60 million hospitalizations that took place between 1999 and 2007 within SUS, showed that geographic areas with more hospitalizations by conditions related to primary health care were those with more private or charitable hospitals and with low coverage of the Family Health Strategy. In contrast, hospitalizations by these conditions were less frequent in areas with greater coverage of the Family Health Strategy and less private or charitable beds^20^. A quality primary health care is an important strategy to avoid unnecessary hospitalizations of older adults with functional limitations.

Few studies, based on national representative samples, examined the social inequalities associated with the use of health services by older adults with functional limitations. In the United States, the relative contribution of functional limitation to doctor visits and hospitalizations is higher among black and Latin people than among white people^1^. An important expression of the social inequalities in Brazil is the access to private health plans, which depend on the capacity of paying^6^. Previous research showed that users of the private system present better health and use more health-care services when compared with users of the public system^6,7^. Our results are in line with these observations. The prevalence of functional limitation was 28% higher among users of the public system when compared with those of the private system. In absolute terms, public system users – with and without functional limitations – performed less doctor visits and were less hospitalized in comparison to private system users. Within the same system, however, our results indicate that the strength of the associations between functional limitation and number of doctor visits and hospitalizations was similar among users of each of these systems.

Due to their form of organization, private health insurances offer more doctor visits with specialists and its users seek care predominantly in doctor’s offices or private clinics^8^. In contrast, the public system offers more doctor visits with general practitioners and the predominant location of the demand for care is the UBS^8^. Our results are consistent with these standards and show that, regardless of the health-care system (public or private), functional limitation is not associated with the specialty of the physician nor with the existence of service or professional of reference. On the other hand, in the private system (but not in the public), we observed greater propensity of older adults with functional limitations to seek UBS, which is a care unit of the public system. The interpretation of this result is not intuitive, since the location sought for care depends on several factors, many of which were not included in this analysis^8,21^. A possible explanation is that the location next to houses or the primary care model adopted by these units is more convenient, in the users’ perception, to the needs of those with functional limitations^d^. Our analysis is limited to examine the issue, but this finding calls attention to the need for deeper investigations for a better understanding of the needs of health care for older adults with functional limitations.

The user’s perception about health care is an important tool for evaluating the health systems^22^. In Brazil, population-based studies based on user’s perception about the care received are still scarce, because until recently there were no nationwide data on the topic^8^. Our results show that, in general, the users’ evaluation of the quality of medical care received was positive. However, this evaluation was a little worse in the public system when compared with the private one, especially by the lack of freedom for choosing the doctor and the long waiting time, in line with the pattern recently described for the whole Brazilian adult population^8^. Our results also show that, among private system users, the evaluation of the seven quality indicators was similar among those with and without functional limitation. In the public system, only two indicators showed association with functional limitation – freedom for choosing the doctor and waiting time for appointment. For both, the prevalence of the evaluation of these indicators as good or very good was lower among those with functional limitation (OR < 1.0).

The main advantage of this study is its large population base, with national representation. On the other hand, this study has limitations inherent to the cross-sectional nature of the research, to the information not included in PNS, and to the provision of data for analysis at the time the study was conducted. In addition, the functional limitation in this study included small difficulty in performing activities of daily living, as well as recent studies conducted in other countries^10^. Therefore, analyses based on the severity of functional limitation may show even stronger associations than those observed in this study.

In short, our results showed strong association between functional limitation, doctor visits, and hospitalizations. Due to the rapid aging of the Brazilian population, an increase in the number of older adults with functional limitations is expected. If effective measures are not extended to health promotion and prevention in their several levels, the increased demand for health care will be inevitable, both for public and private health systems.

## Footnotes

a United Nations, Department of Economic and Social Affairs, Population Division. World population prospects: the 2015 revision. New York; 2015 [cited 2015 Dec 3]. Available from: http://esa.un.org/unpd/wpp

c Instituto Brasileiro de Geografia e Estatística, Coordenação de Trabalho e Rendimento. Pesquisa Nacional de Saúde 2013: acesso e utilização dos serviços de saúde, acidentes e violências: Brasil, grandes regiões e unidades da federação. Rio de Janeiro: IBGE; 2015 [cited 2016 Jan 20]. Available from: http://biblioteca.ibge.gov.br/visualizacao/livros/liv94074.pdf

d Ministério da Saúde (BR), Secretaria de Atenção à Saúde, Departamento de Ações Programáticas e Estratégicas, Área Técnica Saúde do Idoso. Atenção à saúde da pessoa idosa e envelhecimento. Brasília (DF); 2010. (Série B. Textos Básicos de Saúde; (Série Pactos pela Saúde 2006, 12).
